# Soluble urokinase plasminogen activator receptor and lactate as prognostic biomarkers in patients presenting with non-specific chief complaints in the pre-hospital setting – the PRIUS-study

**DOI:** 10.1186/s13049-021-00908-z

**Published:** 2021-08-12

**Authors:** Robert Ivic, Jouni Nurmi, Lisa Kurland, Veronica Vicente, Veronica Lindström, Therese Djärv, Johanna Kaartinen, Maaret Castrén, Katarina Bohm

**Affiliations:** 1 Department of Clinical Science and Education, Södersjukhuset, Karolinska Institutet, Stockholm, Sweden; 2 Academic Emergency Medical Service, Region Stockholm, Stockholm, Sweden; 3 Emergency Medicine, Helsinki University and Department of Emergency Medicine and Services, Helsinki University Hospital, Helsinki, Finland; 4 Department for Medical Sciences, Örebro University, Örebro, Sweden; 5 Karolinska Institutet, Department of Neurobiology, Care Sciences and Society, division of nursing, Stockholm, Sweden; 6 Samariten Ambulance Stockholm, Stockholm, Sweden; 7 Department of Medicine Solna, Karolinska Institutet, Stockholm, Sweden; 8 Department of Emergency Medicine, Södersjukhuset, Stockholm, Sweden

## Abstract

**Background:**

Emergency Medical Services (EMS) are faced daily with patients presenting with non-specific chief complaints (NSC). Patients presenting with NSCs often have normal vital signs. It has previously been established that NSCs may have a serious underlying condition that has yet to be identified. The aim of the current study was to determine if soluble urokinase plasminogen activator receptor (suPAR) and lactate could be used to identify serious conditions among patients presenting with NSCs to the EMS. The secondary aim was to describe the prognostic value for mortality in the group.

**Method:**

A blinded prospective observational cohort study was conducted of patients brought to the ED by ambulance after calling the national emergency number 112 and who were assessed as having NSC by the EMS. Biomarkers were measured during index EMS assessment before transportation to the ED. Patients were followed via EMS and hospital electronic health records. Descriptive and logistic regression analyses were used.

**Results:**

A total of 414 patients were included, with a median age of 82 years. A serious condition was present in 15.2% of the patients. Elevated suPAR above 3 ng/ml had a positive likelihood ratio (LR+) of 1.17 and a positive predictive value (PPV) of 17.3% as being predictive of a prevalent serious condition. Elevated suPAR above 9 ng/ml had LR+ 4.67 and a PPV of 16.7% as being predictive of 30-day mortality. Lactate was not significantly predictive.

**Conclusion:**

Pre-hospital suPAR and lactate cannot differentiate serious conditions in need of urgent treatment and assessment in the ED among patients presenting with non-specific chief complaints. suPAR has shown to be predictive of 30-day mortality, which could add some value to the clinical assessment.

**Trial registration:**

NCT03089359. Registered 20 March 2017, retrospectively registered, https://clinicaltrials.gov/ct2/show/NCT03089359.

## Background

Emergency Medical Services (EMS) play an important role in assessing, initiating treatment and if needed, transporting patients to the Emergency Department (ED).

Patients presenting with non-specific chief complaints (NSC) are often assessed as having an “affected general health condition” or “decreased general condition”, “a general malaise”, “a sense of illness”, or “just being unable to cope with usual daily activities”, and often present with near normal vital signs [1–3]. It is not known how many present to the EMS with NSCs, although NSC’s have been studied in the pre-hospital setting and a majority of the NSCs arrive at an ED by ambulance [4, 5] and most often during the daytime [5]. One in three patients presenting to the EMS with NSCs have serious conditions [6]. Previous studies have shown that up to one in five patients in the ED have NSCs and that half of these patients are suffering from an underlying serious condition [7, 8]. Patients presenting with NSCs are often elderly, and as many as half of these patients suffer from an acute condition [9]. The elderly presenting with NSCs are often under-triaged [9], despite having the highest in-hospital mortality rate of all non-trauma/non-surgical chief complaints in the ED [10]. Vital-sign-based triage systems may be insufficient when attempting to identify patients at high risk of having a serious condition and of dying among those presenting to the EMS with NSCs [6, 11]. In addition to the triage systems used, biomarkers could be a feasible supplemental tool.

Although disease and serious conditions may have a broad range of causes, the physiological response is relatively uniform. The severity of the disease is determined by the degree of systemic inflammation and subsequent hemodynamic changes, the extent of biological stress, organ failure, and ultimately death. Therefore, circulating mediators of core pathways may potentially serve as prognostic biomarkers [12].

Soluble urokinase plasminogen activator receptor (suPAR) is released during inflammation or immune activation. The suPAR level can be used as a biomarker reflecting the extent of immune activation in the individual [13]. The suPAR level is elevated across diseases, and not solely associated with one specific disease. Studies have shown that the suPAR level is associated with morbidity and mortality [14–22]. suPAR has been shown to have prognostic values in patients presenting with chest pain to the ED, deterioration of patients with suspected bacterial infection, as well as a decision marker for early discharge of patients with COVID-19 symptoms [23–25]. However, the diagnostic properties of suPAR compared to C-reactive protein (CRP) and interleukin-6 (IL-6) are weak in infectious diseases [17]. Therefore, suPAR could be applicable as a prognostic marker for serious conditions in patients presenting with NSCs, before deviated vital signs appear. This characteristic may be utilized for risk stratification in unselected patients.

Lactate serum concentration reflects the balance between lactate production and lactate consumption. Elevated values reflect an increased anaerobic metabolism secondary to hypoperfusion in tissue, with several etiologies [26, 27]. Lactate as an indicator of hypoperfusion in tissue prior to the development of clinical findings may serve as a prognostic, risk-stratifying biomarker. It has been suggested as having prognostic properties also in the pre-hospital setting by previous studies [28, 29].

In order to increase patient safety, and to attempt to identify serious conditions among patients presenting with NSCs, additional assessment tools are required. Therefore, the aim of this study was to determine if suPAR and lactate could be used to identify serious conditions among patients presenting with NSCs to the EMS. The secondary aim was to describe the prognostic value for mortality in the group.

## Method

### Study design

We performed a blinded prospective observational cohort study of patients brought to the ED by ambulance after calling the national emergency number 112 and who were assessed as having an NSC by the EMS.

### Settings and population

The Pre-hospital Recognition and Identification of Unspecific Symptoms (PRIUS) study was initiated in May 2015 and completed in September 2017 and was carried out in Stockholm Region, Sweden and Uusimaa Region, Finland.

Stockholm Region had a population of approximately 2.1 million (year 2015). The Region was responsible for operating the EMS, and the service was provided by one organization within the region and two private companies. The EMS in Stockholm had almost 190,000 assignments in 2016. The number of ambulances in the area was 71 during the daytime and 40 during the night. All ambulances in Stockholm, Sweden were manned by a nurse specialist and an emergency medical technician (EMT).

The Uusimaa Region participating in the current study in Finland had a population of 480,000. EMS in Uusimaa Region were organized by Helsinki University Hospital and provided by two fire departments and two private companies, operating 21 ambulances. The annual assignment rate was 50,000. In the Finnish EMS there are two levels of ambulances. Basic life support (BLS) units are staffed by two crew members with the minimum training requirement of vocational qualification in health care specialized in emergency care. The other member of the crew can be a health care professional (eg. a qualified nurse) or a fire fighter. In an Advanced life support (ALS) unit at least one of the crew must have a bachelor’s degree in prehospital care or a degree in nursing with an additional specialization course in prehospital care. The other member of the crew can be either a health care professional or a fire fighter.

### Study participants/ population

In the current study, patients presenting with NSCs to the EMS were included. The NSCs were defined as a presenting complaints of “decreased general condition”, “fatigue”, “malaise”, or “feeling unwell” upon EMS arrival.

The inclusion criteria were NSCs, Swedish or Finnish identification numbers respectively, being 18 years of age or above, informed consent by the patient or if personally unable, next of kin on behalf of the patient, transportation to an ED in Stockholm or Helsinki respectively, and normal vital signs, defined as: a heart rate of 50–110 beats/minute, oxygen saturation over 90%, systolic blood pressure over 100 mmHg, a respiratory rate 10–25 respirations/minute, body temperature of 36.0–38.5 C, and Glasgow Coma Scale [GSC] 15***.*** The exclusion criteria were not meeting inclusion criteria in the case of simultaneous specific complaints, the patient was not eligible for inclusion.

### Diagnostic tests

After informed consent patients were enrolled in the study by the EMS-personnel, a peripheral venous cannula was inserted, and two blood samples were obtained before being transported to the ED. The samples were sent to the laboratory at the receiving hospital.

Samples for lactate analyzed continuously as a routine analysis, using an enzyme-based colorimetric assay (Roche Diagnostics Scandinavia AB, Solna, Sweden). Samples for suPAR were analyzed using a commercial enzyme immunoassay (ViroGates, Birkeroed, Denmark). EMS and receiving hospitals were blinded to test-results, in order not to alter or affect the assessment according to current EMS guidelines or the patient protocols at each ED.

### Data collection

Electronic health records were obtained for all patients enrolled in the study from CAK-net [30] and Take Care (CGM, Stockholm, Sweden) in Sweden and Merlot Medi (CGI Finland, Helsinki, Finland) in Finland. In-hospital patient data were obtained from electronic health records. The data collection included the following components for each patient: age, sex, vital signs at EMS triage, ED discharge diagnosis according to International Classification of Disease (ICD) 10th revision, ED discharge disposition (home, admission to hospital), length of stay (LOS) in hospital if admitted, discharge diagnosis (ICD-10), admission to in-hospital care, mortality (24 h and 30 days), and Charlson Comorbidy Index [31], calculated by the researchers based on the patients’ record data.

### Outcomes

The primary outcome in the current study was a serious condition, as previously defined for EMS purposes [6].

### Statistical analysis

Descriptive statistics were used. Continuous variables are presented as median and interquartile range [IQR], while proportions are presented as percentages. Differences between groups were evaluated using a Chi^2^- test and Fisher’s exact test, where appropriate, for categorical variables and a Mann-Whitney U Test for numerical variables. Logistic.

regression analysis was performed to assess the association of serious conditions and the mortality rates after the index assessment by the EMS. Area under receiver operating characteristics (AUROC) was calculated to assess the accuracy of the biomarkers tested. The prediction-model was based on suPAR, age, and sex for absolute risk prediction. Positive and negative likelihood ratios as well as positive predictive values (PPV) and negative predictive values (NPV) were calculated from 2 × 2 contingency tables (crosstabulation) based on the diagnostic test and the outcome for the biomarkers. Before analysis cut-off, values for suPAR were defined as ≥3, ≥6, and ≥ 9 ng/ml based on [32, 33] The cut-off for lactate was defined as ≥2.3 mmol/l based on the reference standard from Karolinska University Laboratory in Stockholm, Sweden. All statistical analysis was performed using IBM SPSS Statistics for Windows, version 25 (IBM Corp. Armonk, New York, USA).

## Results

**Fig. 1:**
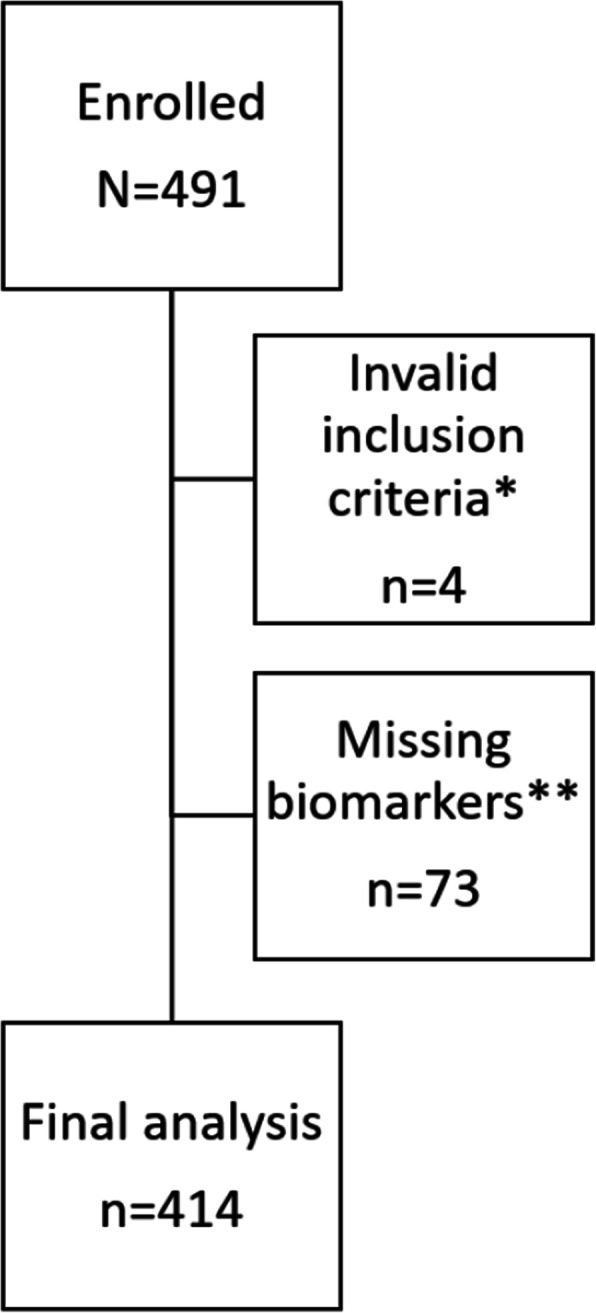
Flow chart over patients included in the study. *: Vital signs not within range. **: Missing laboratory test results of suPAR and/or lactate

A total of 491 patients were enrolled in the current study and 414 of these were included in the final analysis (Fig. 1). The median age was 82 (IQR 75–88) years of age and 56.5% (*n* = 234) of these were female. The overall admission rate to in-hospital care was 55.1% (*n* = 225) with a median in-hospital LOS of 3 days (IQR 0–9) (Table 1).

**Table 1:** Patient characteristics and outcome

		Total			Serious condition			Serious condition		
					**present**			**not present**		
		*N* = 414			*n* = 63 (15.2%)			*n* = 351 (84.8%)		
		n	Md (IQR)	(%)	n	Md (IQR)	(%)	n	Md (IQR)	(%)
Sex	Female	234		(56.5)	34		(54.0)	200		(57.0)
	Male	180		(43.5)	29		(46.0)	151		(43.0)
Age	Md		82 (75–88)			85 (79–90)			81 (75–88)	
Admitted	Yes	225		(54.3)	54		(85.7)	171		(48.7)
	No	189		(45.7)	9		(14.3)	180		(51.3)
In-hospital LOS			2 (0–9)			8 (3–13)			1 (0–7)	
CCI			2 (0–9)			1 (0–2)			1 (0–7)	
24 h mortality		0		(0.0)	0		(0.0)	0		(0.0)
30 day mortality		17		(4.1)	6		(9.5)	11		(3.1)

*LOS: length of stay. CCI: Charlson comorbidity index;*

A serious condition was present in 15.2% (*n* = 63) of the included patients. The absolute risk for having a serious condition was at the highest at 34.9% for men older than 80 years and having a suPAR above 9 ng/ml. Overall 30-day mortality was 4.1% (*n* = 17). In the group with serious conditions, 30-day mortality was 9.5% (*n* = 6), compared to 3.1% (*n* = 11) in the group with no serious conditions (Table 2).

**Table 2:** Biomarkers for serious condition by 30-day mortality

		Total *N* = 414						Serious condition Present *n* = 63						Serious condition Not present *n* = 351					
		Alive *n* = 397			Deceased *n* = 17			Alive *n* = 57			Deceased *n* = 6			Alive *n* = 340			Deceased *n* = 11		
		n	Md (IQR)	(%)	n	Md (IQR)	(%)	n	Md (IQR)	(%)	n	Md (IQR)	(%)	n	Md (IQR)	(%)	n	Md (IQR)	(%)
suPAR	ng/ml	4.8 (3.5–6.6)			10.7 (5.8–13.3)			5.4 (4.4–7.4)			12.5 (5.8–15.2)			4.7 (3.5–6.6)			10.3 (4.5–12.7)		
suPAR	0–3.9	56		(14.1)	0		(0.0)	1		(1.8)	0		(0)	55		(16.2)	0		(0.0)
	4.0–5.9	211		(53.1)	5		(29.4)	33		(57.9)	2		(33.3)	178		(52.4)	3		(27.3)
	6.0–8.9	80		(20.2)	2		(11.8)	14		(24.6)	0		(0)	66		(19.4)	2		(18.2)
	≥9	50		(12.6)	10		(58.8)	9		(15.8)	4		(66.7)	41		(12.1)	6		(54.5)
Lactate	mmol/l	1.7 (1.3–2.3)			1.8 (1.7–3.3)			1.6 (1.3–2.0)			1.8 (1.7–2.0)			1.7 (1.3–2.4)			2.8 (1.6–4.9)		
Lactate	≤2.2	288		(72.5)	10		(63.6)	45		(78.9)	5		(83.3)	243		(71.5)	5		(45.5)
	≥2.3	109		(27.5)	7		(36.4)	12		(21.1)	1		(16.7)	97		(28.5)	6		(54.5)

*suPAR: soluble urokinase plasminogen activator receptor*

**Fig. 2:**
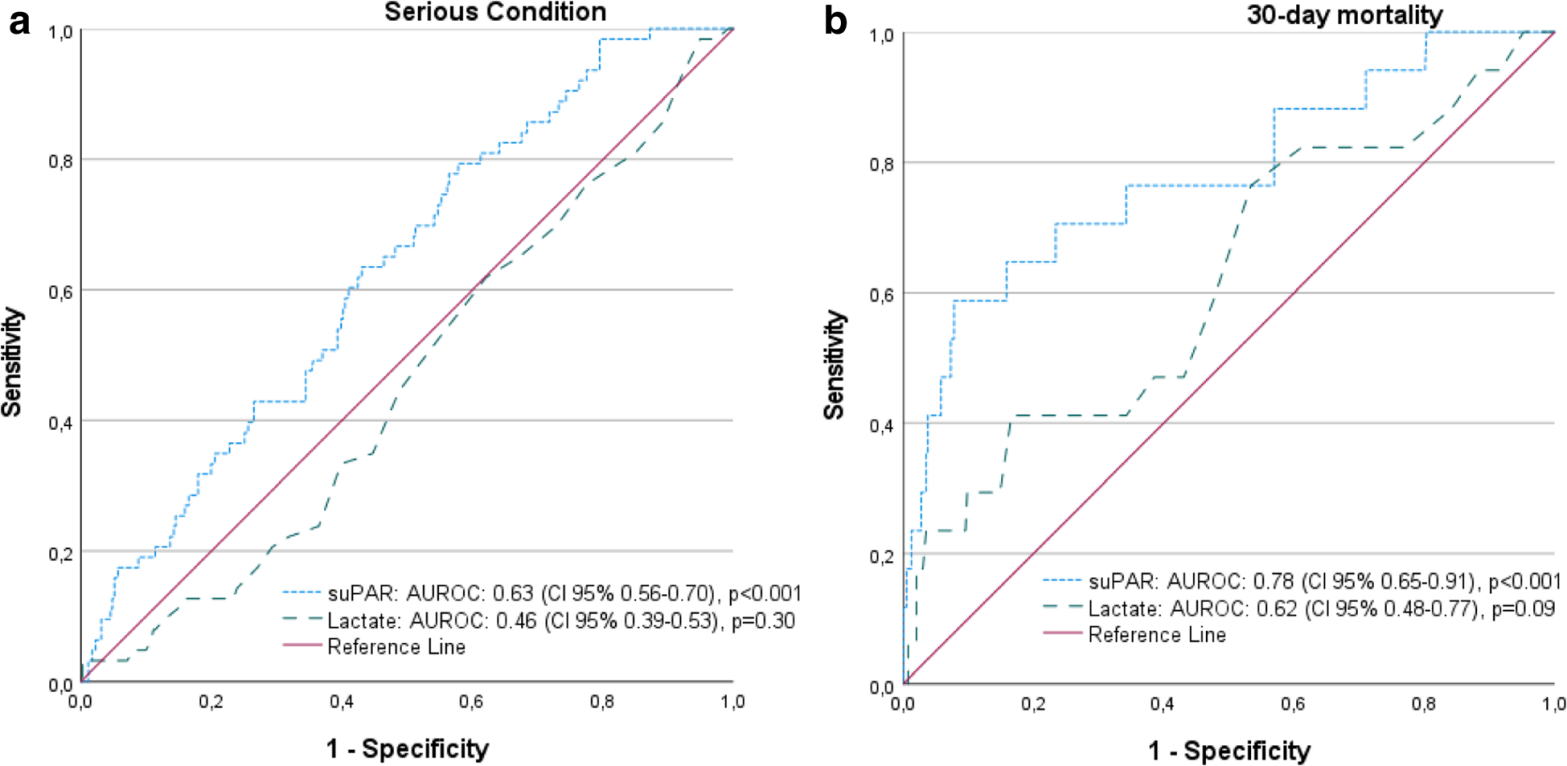
**A**: ROC curve based on suPAR and lactate by prevalent serious condition. **B**: ROC curve based on suPAR and lactate by 30-day mortality

The area under receiver operating characteristics (AUROC) for suPAR and lactate for having a serious condition was 0.63 (95% CI 0.56–0.70), *p < 0.001 and* 0.46 (95% CI 0.39–0.53), *p = 0.30* respectively. The AUROC for suPAR and lactate and 30-day mortality was 0.78 (95% CI 0.65–0.91), *p < 0.001* and 0.62 (95% CI 0.48–0.77), *p = 0.09 (*Figs. 2a, 2b).

suPAR above 3 ng/ml had a positive likelihood ratio (LR+) of 1.17 and a positive predictive value (PPV) of 17.3%, predicting a serious condition. suPAR above 9 had LR+ 4.59 and a PPV of 16.1% of predicting 30-day mortality. Lactate was not significantly predictive of serious conditions or 30-day mortality (Tables 3 and 4).

**Table 3:** Predictive ability of soluble urokinase plasminogen activator receptor (suPAR) and lactate with respect to serious conditions and 30-day mortality. *P* value < 0.05 is considered significant

		Serious condition *n* = 414			30 day mortality *n* = 414		
		*PPV*	*NPV*	*sig.*	*PPV*	*NPV*	*sig.*
suPAR	≥3 ng/ml	17.3%	98.2%	*p < 0.001*	4.7%	100%	*p = 0.145*
	≥6 ng/ml	19.0%	86.8%	*p = 0.08*	8.5%	98.2%	*p = 0.03*
	≥9 ng/ml	21.7%	85.9%	*p = 0.171*	16.7%	98.0%	*p < 0.001*
Lactate	≥2.3	11.2%	83.2%	*p = 0.173*	6.0%	96.8%	*p = 0.269*

**Table 4:** Likelihood ratios of soluble urokinase plasminogen activator receptor (suPAR) and lactate being predictive for serious conditions and 30-day mortality. P value < 0.05 is considered significant

		Serious condition *n* = 414			30 day mortality *n* = 414		
		*+LR*	*-LR*	*sig.*	*+LR*	*-LR*	*sig.*
suPAR	≥3 ng/ml	*1.17*	*0.0*	*p < 0.001*	*1.16*	*0*	*p = 0.145*
	≥6 ng/ml	*1.31*	*0.91*	*p = 0.08*	*2.16*	*0.43*	*P = 0.003*
	≥9 ng/ml	*1.54*	*0.92*	*p = 0.171*	*4.67*	*0.47*	*p < 0.001*
Lactate	≥2.3	*0.70*	*1.12*	*p = 0.173*	*1.50*	*0.81*	*p = 0.269*

## Discussion

The biomarkers suPAR and lactate cannot identify serious conditions among patients presenting with NSCs to the EMS. We observed an association between prehospital measured suPAR and lactate with the presence of a serious condition, however, the accuracy of these biomarkers to differentiate serious conditions in need of urgent treatment and assessment in the ED among patients presenting with a non-specific chief complaint is too low for clinical use in the EMS setting.

The results show that one in six patients presenting with NSCs had a serious condition. Compared to previous studies in the pre-hospital setting, the numbers are comparable [6], and lower compared to in-hospital studies [8, 34, 35]. The predictive value of the tests for serious conditions was evaluated by the likelihood ratios presented and by the positive predictive values which were indicative of a rarely useful test. Although most of the tests were not statistically significant, the low values confirm the results, that these biomarkers are of little to no use in identifying the serious conditions among patients presenting with non-specific chief complaints to the EMS. Even though AUROC suggests that suPAR may perform well as a predictive test for serious conditions, the area under the curve was merely moderate at most. The highest absolute risk was around 35% for the oldest patient with the highest suPAR, which further strengthens the finding that suPAR is of little to no use in identifying the serious conditions among patients presenting with NSCs to the EMS.

Mortality rates were three times higher in the group with serious conditions as compared to those without serious conditions. The predictive value of suPAR on 30-day mortality was elevated, indicating that the test could sometimes be useful, while the positive predictive values were not convincing. Elevated suPAR levels have shown to be predictive for mortality, as previously reported [18, 36, 37], but not for serious condition. The predictive value for mortality could be an indicator for further medical assessment and risk stratification since it is associated with mortality in general but not specifically for patients with non-specific chief complaints.

No patient died within the first 24 h of the current study; hence, one could discuss whether the ED is an appropriate level of care and medical assessment. On the other hand, the high admittance rate to in-hospital care in the group with serious conditions suggests that the indication for transporting to an ED is strong. Identification of patients with serious conditions among those with NSCs in the prehospital setting remains a challenge. The biomarkers in the current study have shown to be unable to differentiate between patients with serious conditions and those without serious conditions.

### Limitations

Patients eligible for inclusion in the current study were patients who presented with NSCs and vital signs within normal range. This group of patients may after assessment be directly referred to geriatric care unless they are below 65 years of age, without passing the ED. All patients can choose to refrain from further assessment in the ED, irrespective of the recommendation by EMS. Direct admission to geriatric care or non-conveyed patients were not included since one of the inclusion criteria was being transported to an ED. Therefore, consecutive inclusions were not possible. The EMS personnel and staff at the receiving hospital was blinded from the biomarker test results measured in the ambulance. If not blinded as in this study, suPAR and lactate results could potentially have affected the EMS-personnel and/or hospital staff into assessments based on the biomarkers, even though their significance and predictive values were not yet evaluated.

The result of suPAR and lactate being poor prognostic biomarkers for identifying serious conditions are generalizable only for patients presenting with NSCs to the EMS and having vital signs within the normal range.

### Clinical implications and further research

The aim of the current study was to determine whether serious conditions could be identified by suPAR and lactate as supplements to vital-signs-based triage. Since triage models in this current study are based on vital signs and patient history, patients with an absence of specific symptoms and presenting with normal vital signs, will be triaged to the lowest category, and will wait longer periods for assessment by a physician. If they have an underlying serious condition, the waiting time could be detrimental. Serious conditions could not be identified by suPAR and lactate in the group of patients presenting with NSCs to the EMS.

## Conclusion

The results show that pre-hospital soluble urokinase plasminogen activating receptor (suPAR) and lactate are not predictive of serious conditions among patients presenting with non-specific chief complaints. One in six patients presenting to the EMS with NSCs have a serious condition. The presence of serious conditions is associated with a three times higher 30-day mortality rate compared to patients without serious conditions. suPAR has shown to be predictive of 30-day mortality, which could potentially add value to the clinical assessment.

## Data Availability

Anonymized data analyzed for the current study will be shared if a reasonable request is made by a qualified investigator to the corresponding author.

## Abbreviations

ALS: Advanced Life Support
AUROC: Area under receiver operating characteristics
BLS: Basic Life Support
CCI: Charlson Comorbidy Index
ED: Emergency Department
EMS: Emergency Medical Services
EMT: Emergency Medical Technician
ICD10: International Classification of Disease (ICD) 10th revision
IQR: Interquartile Range
LOS: Length of stay
LR +: Positive Likelihood Ratio
NPV: Negative Predictive Value
PPV: Positive Predictive Value
PRIUS: Pre-hospital Recognition and Identification of Unspecific Symptoms
suPAR: Soluble urokinase plasminogen activating receptor
